# Skull Vibration-Induced Nystagmus and High Frequency Ocular Vestibular-Evoked Myogenic Potentials in Superior Canal Dehiscence

**DOI:** 10.3390/audiolres12020023

**Published:** 2022-04-14

**Authors:** Ángel Batuecas-Caletrío, Alejandra Jara, Victor Manuel Suarez-Vega, Susana Marcos-Alonso, Hortensia Sánchez-Gómez, Nicolas Pérez-Fernández

**Affiliations:** 1 Otoneurology Unit, Department of Otorhinolaryngology, Complejo Asistencial Universitario de Salamanca, IBSAL, University of Salamanca, 37004 Salamanca, Spain; susana.ma.95@gmail.com (S.M.-A.); hortensiasanchez1@hotmail.com (H.S.-G.); 2 Department of Otorhinolaryngology, Hospital General Universitario Reina Sofía, 30005 Murcia, Spain; alexandera.jm23@gmail.com; 3 Department of Otorhinolaryngology, Hospital Universitario Morales Meseguer, 30005 Murcia, Spain; 4 Department of Radiology, Clínica Universidad de Navarra, 28015 Madrid, Spain; vvega@unav.es; 5 Department of Otorhinolaryngology, Clinica Universidad de Navarra, 28015 Madrid, Spain; nperezfer@unav.es

**Keywords:** superior canal dehiscence, skull vibration-induced nystagmus, SVINT, ocular vestibular-evoked myogenic potentials, HFoVEMPS, vestibular disorders

## Abstract

Background: Although diagnostic criteria have been established for superior canal dehiscence syndrome, cases in which the diagnosis is not easy are frequent. On those occasions, some tests such as vibration-induced nystagmus or vestibular-evoked myogenic potentials can offer invaluable help due to their high sensitivity and specificity. Methods: We studied 30 patients showing superior canal dehiscence or “near-dehiscence” in a CT scan. Skull vibration-induced nystagmus and high frequency ocular vestibular-evoked myogenic potentials are performed in each patient. The aim of the study is to determine how useful both tests are for detection of superior canal dehiscence or near-dehiscence. Results: Of the 60 temporal bones studied, no dehiscence was the result in 22, near-dehiscence in 17 and a definite finding in 21. In 10/30 patients, there was no SVIN (Skull vibration induced nystagmus) during otoneurological testing, while in 6/30, induced nystagmus was mainly horizontal, and in 14/30 there was vertical up-beating. All patients had a positive oVEMP (Ocular vestibular evoked myiogenic potentials) at 0.5 kHz in both ears and the HFoVEMP (High frequency ocular vestibular evoked myiogenic potentials) response was positive in 25/60 (41.6%) of the ears studied and in 19/30 of the patients evaluated (in 6 it was positive in both ears). Up-beat SVIN will point to a SCD (Superior Canal Dehiscence) mainly when HFoVEMP are present, and when this is negative there is a high probability that it is not a SCD. Conclusions: When SVIN and HFoVEMP results are added (or combined), they not only improve the possibilities of detecting SCD, but also the affected side.

## 1. Introduction

Superior canal dehiscence syndrome (SCD) was first described by Minor et al. in 1998 [1]. Patients suffering from chronic disequilibrium and sound- or pressure-induced vertigo, who had nystagmus in the plane of the superior semicircular canal and a computed tomography revealing a bony dehiscence over the superior semicircular canal were considered. Other additional symptoms such as pulsatile tinnitus or hyperacusis to bone conducted sounds were also included in the syndrome’s initial report.

The etiology of SCD is unclear as well as its pathophysiology. Congenital or acquired conditions have been studied but none of them could completely explain all the cases of SCD [2,3]. Pathophysiologically, the relationship between a bony dehiscence over the superior semicircular canal and the symptoms in SCD was explained by a third mobile window in the labyrinth, altering the biomechanics of the inner ear [4].

The third mobile window model could explain frequent findings in SCD patients of low-frequency conductive hearing loss and negative bone conduction thresholds on pure tone audiometry, lower thresholds for cervical vestibular-evoked myogenic potentials (VEMPs), higher amplitude responses for ocular VEMPs and the eye movements, which can be observed in the plane of the superior semicircular canal, that are induced by sound or pressure directly applied to the ear canal or by a Valsalva maneuver [5].

In fact, the low-frequency conductive hearing loss and the air-bone gap with an intact stapedial reflex is the main difference with other low-frequency hearing loss etiologies such as otosclerosis [6].

Not all patients with an anatomic dehiscence in the superior semicircular canal show SCD symptoms and not all symptoms mimicking SCD (aural fullness, low-frequency conductive hearing loss and negative bone conduction thresholds on pure tone audiometry or eye movements induced by sound) can be associated with a superior semicircular canal dehiscence. Consensus diagnostic criteria were important to get a correct diagnosis [5].

Since the first description of SCD, the efforts towards a better understanding of the clinical and examination findings have been continuous and the skull vibration-induced nystagmus (SVIN) has not been an exception [7].

Given that a bone-conducted vibration induces an activation in the canal afferents of both labyrinths [8], the effect of SVIN in SCD patients has been studied for years [7].

A 100-Hz bone-conducted vibration applied to either mastoid instantaneously induces a predominantly horizontal nystagmus, with quick phases beating away from the affected side in patients with a total unilateral vestibular loss and in almost all of the patients with partial unilateral vestibular loss [9,10].

This test is a robust indicator of the asymmetry of vestibular function in vestibular neuritis [11], intratympanic gentamicin [12] or unoperated vestibular schwannoma patients [13], but it is not useful in Meniere disease or SCD patients, in which the results of SVIN are frequently controversial and not aligned with the general criteria for total unilateral vestibular loss [9].

One frequent finding in unilateral SCD with the SVIN test is a nystagmus with torsional and horizontal quick phases beating towards the lesion side [7]. Moreover, a primarily down-beating nystagmus is preferentially observed in patients when the gaze is directed towards the plane of the superior dehiscent canal [14].

The aim of this study is to describe the SVIN and High Frequency ocular VEMP (HFoVEMP) findings in a cohort of SCD patients with a temporal bone CT scan that proved superior canal dehiscence.

## 2. Methods

### 2.1. Patients

We performed a retrospective study. We included 30 patients: 18 female and 12 male. All of them were seen for intermittent dizziness or chronic disequilibrium. They are all part of an individual database of 1183 patients which represents 2.5% of those seen in a dedicated otoneurologic clinic. All of the patients were diagnosed after a CT scan evaluation (read later) of SCD or near-dehiscence (Near D). Exclusion criteria were concomitant ear disease (chronic otitis media, otosclerosis) or vestibulopathy (Ménière’s disease, vestibular migraine or primary BPPV (Benign Paroxysmal Positional Vertigo)). All of them were tested at bedside and in a vestibular laboratory; in particular, the SVIN test and the air conducted ocular vestibular-evoked myogenic potentials were performed the same day.

### 2.2. SVIN

#### 2.2.1. Stimulation

As it is described in [15], SVIN was evoked with the patient in a sitting position by stimulating both mastoid processes and the vertex with three stimulation sequences for 10 s using a 100 Hz handheld vibrator (VVIB 100; Synapsys, France), with 15 s breaks between each stimulation. Evoked nystagmus upon stimulation was recorded using videonystagmography in a dark room and in a vision-denied setting. Baseline eye movement was recorded prior to stimulation of the mastoid processes. Subjects were instructed to continue looking straight ahead while stimulation was applied for approximately 10 s. The Slow Phase Velocity (SPV) of the horizontal and vertical components of the SVIN were obtained by calculating the slope of the slow-phase eye movement in the 10 s window using VNG software (Ulmer, Marseille, France). In this study, the torsional component of the nystagmus was not measured and may constitute a limitation. However, in clinical usual practice, physicians use only 2D recordings and the purpose was to know whether an analysis limited to vertical and horizontal components could bring sufficiently contributive data.

Validity criteria for a positive SVIN were [15]:

1. VIN starts and stops with stimulation, does not present any secondary reversal, is constant on both mastoid processes, and beats in the same direction.
2. The slow-phase velocity (SPV) of the VIN as measured in the horizontal canal must be >2.5°/s.
3. It is reproducible and must be identical or similar on two successive tests.

#### 2.2.2. VEMP Testing

Stimulation mode: 0.5 kHz, 1 khz and 4 kHz air-conducted tone bursts were presented monaurally through a pair of calibrated ABR3A insert earphones at an intensity of 97 dB nHL. In this work, we shall consider in particular the results from 4 kHz or high frequency HFoVEMP. A Blackman envelope was configured (rise/fall time 2 ms, plateau time 0 ms). In total, 100 averages were presented at a rate of 5.1/s.

*Location of the electrodes and registration:*

The VEMPs were registered with the ICS (Chartr, Otometrics, Taastrup, Denmark). For oVEMPs, the surface registration electrode was placed on the skin just below the margin of the eyelid contralateral to the stimulated ear and the reference electrode immediately 2 cm further down on the same cheek. The earth electrode was placed on the lower forehead. The impedances were kept below 3 kHz. They were recorded with patients sitting upright with their heads forward. They were instructed to look at a fixed point on the wall (approximately 1.5 m away) with an upwards inclination of 35°. The potentials were recorded from 20 ms before the start of the stimulation until 80 ms later.

VEMP response. The response evoked by oVEMP presents a negative (n10) and positive (p16) wave. For this study, we analyzed the latencies and the amplitudes of the responses after the stimulation of the right and left ears.

VEMP calculation of amplitude. The number of recordings made per subject was based on the reproducibility of the observed response. In the cases in which the response was absent, the mean amplitude was considered null (0 µV).

#### 2.2.3. CT Scan

All patients underwent a high-resolution CT (HRCT) scan of the temporal bones performed at a dual source CT scanner (Somatom Drive, Siemens Heatlthineers, Erlangen, Germany). Images were obtained with a slice thickness of 0.5 mm [16], spacing of 0.25 mm, pitch of 0.85, 130 kV (peak), mAs with automated exposure control in order to minimize radiation dose, and bone and soft tissue kernels of reconstruction. Studies were obtained in the axial plane and reformatted to the coronal plane. Additionally, oblique Poschl views were obtained in all studies in order to assess the presence of SCD more confidently [17].

Scans were reviewed by one radiologist with years of experience in head and neck imaging.

The superior semicircular canal status was characterized as Normal (patent bony coverage could be clearly visible), frank or Definite dehiscent (no bony coverage at some point of the superior canal) or near-dehiscent (Near D) (marked thinning of the bony coverage, but still present and visible on CT scans).

#### 2.2.4. Statistics

The statistical analyses were performed using SPSS (version 19, SPSS Inc., Chicago, IL, USA) statistical software. To perform the descriptive analysis of the amplitudes of the VEMPs, we used mean ± standard error. Contingency tables were created for qualitative variables and the chi-square test was used. All tests were 2-tailed, and *p* values of 0.05 were considered to be statistically significant.

The findings in the CT scan were reconsidered and 2 groups were created: (1) SCD+ when in any of the ears or in both there was a positive finding of SCD, and (2) SCD−, when in any or in both there was a near-dehiscence (Near D) and in the other there was no SCD.

## 3. Results

The mean age of the 30 patients included here was 54 ± 15 years.

### 3.1. SCD Finding in CT Scan

**Figure 1:**
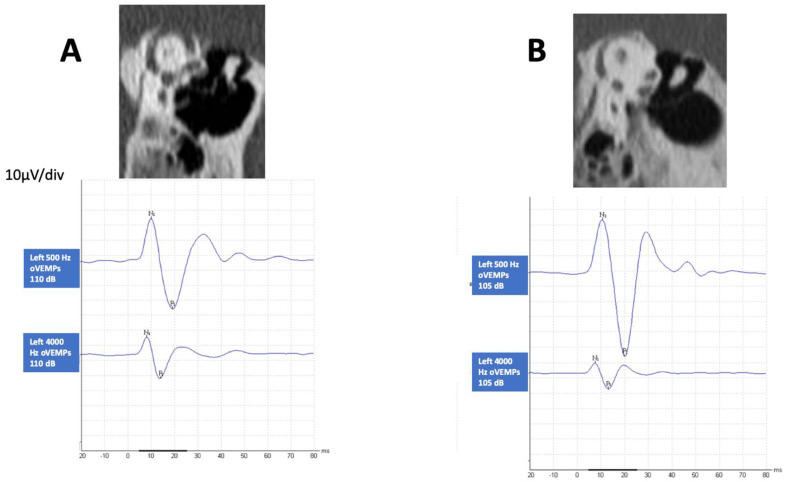
CT scan finding of two patients that shows a left definite dehiscence (**A**) and left near-dehiscence (**B**) with the corresponding oVEMP findings at 0.5 kHz and 4 kHz air-conducted sound stimulating each ear.

All patients had some degree of SCD in either or both ears according to radiology inclusion criteria. In 13 (43%) patients, one of the temporal bones was normal, and in the other, there was a near-dehiscent SSC (Superior Semicircular canal); in 9 (30%) patients, one side was normal, and in the other, there was a clear dehiscent SSC; in 5 (16.6%) patients, in both sides the SSC was dehiscent; in 2 (6.6%) patients, one side was dehiscent and the other near-dehiscent; in 1 (3.3%) patient, both sides were near-dehiscent. As shown in Table 1, bilateral SCD was found in 5 patients, and unilateral in 11 patients. Of the 60 temporal bones studied, no dehiscence was found in 22, near-dehiscence in 17 and a definite finding in 21. When a more precise classification was done, there were 14 patients in the SCD group and 16 in the SCD+ group; in 11, SCD was unilateral, and in 5, bilateral. In Figure 1, we present the CT scan image of two different cases showing both types of abnormality and the corresponding HFoVEMP.

### 3.2. SVIN

In 10/30 patients, there was no SVIN during otoneurological testing, while in 6/30, induced nystagmus was mainly horizontal, and in 13/30, vertical up-beating. The mean SPV of SVIN in the former was 4.4 ± 1.8°s^−1^ and in the latter 1.9 ± 0.6°s^−1^. Given that some degree of dehiscence had to occur, this represents that in 2/3 patients with SSCD there is some degree of SVIN. According to the type of finding in the temporal bones, the SVIN obtained is shown in Table 2.

**Figure 2:**
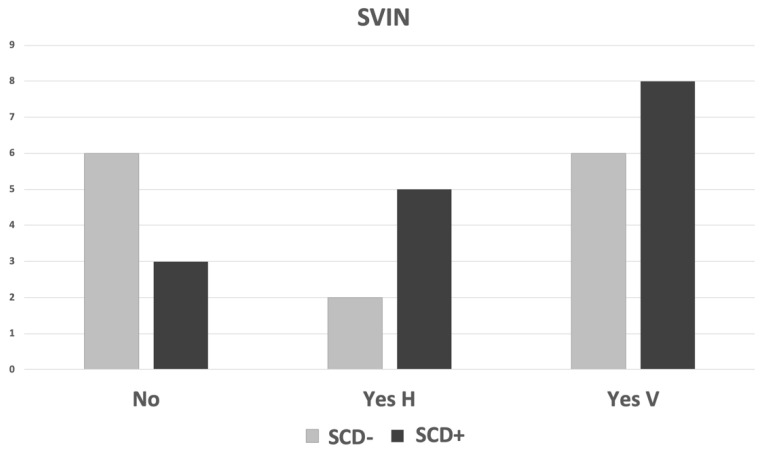
Type of SVIN and findings in the CT scan by patient (N = 30). Near-dehiscence was considered as no-dehiscence (SCD−) for analytical purposes. H: horizontal induced SVIN; V: vertical up-beating SVIN.

We first analyzed SVIN to detect SCD in patients (groups SCD and SCD+) and we found that the specificity of the SVIN to detect a patient with SCD is 0.43 and its sensitivity is 0.81: the positive predictive value is 0.62 and the negative predictive value is 0.67. In a more detailed way, we then searched for the type of nystagmus, and the CT scan findings and results are shown in Figure 2. There is no association between SVIN detection and SCD group (chi-square = 4.438, *p* = 0.108) when all SCD are considered (both uni- and bilateral patients).

### 3.3. HFoVEMP

All patients had a positive oVEMP at 0.5 kHz in both ears, and the HFoVEMP response was positive in 25/60 (41.6%) of the ears studied and in 19/30 patients evaluated (in 6 it was positive in both ears).

**Figure 3:**
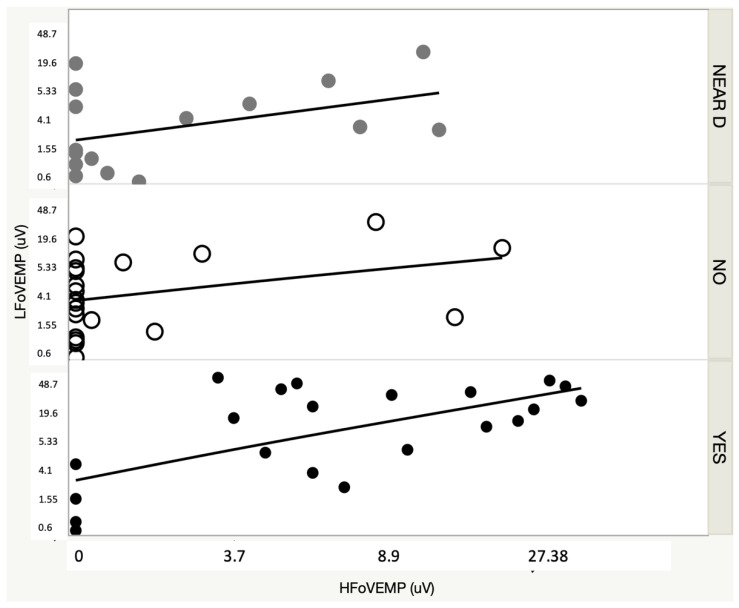
HFoVEMP and LFoVEMP amplitudes with regard to the CT scan finding. Near D: near-dehiscence; NO: no dehiscence; Yes: dehiscence.

The mean amplitude of the n10-p16 response was 1 ± 1.6 µV in the group in which the response was considered doubtful and 11.9 ± 8.6 µV in the group in which the response was considered positive. In Figure 3, we present the amplitude of the HFoVEMP to the result obtained in the 0.5 kHz OVEMP (LFoVEMP) with regard to the finding in the CT scan.

**Figure 4:**
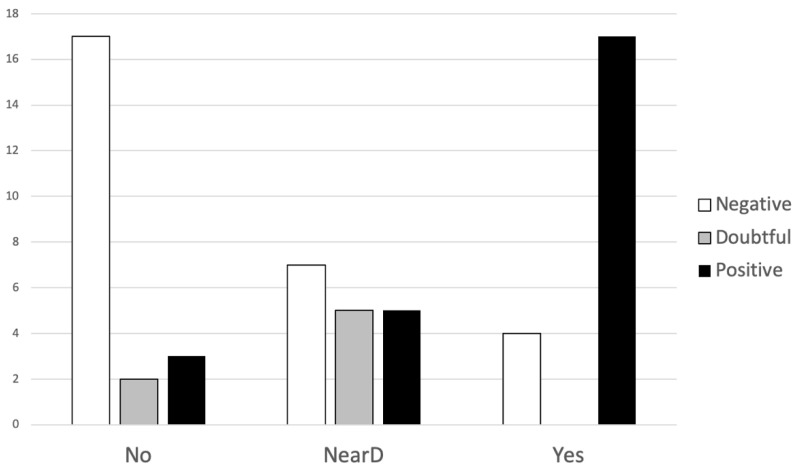
Type of HFoVEMP to superior semicircular canal finding in CT scan. No: normal CT, NearD: near-dehiscence, Yes: definite SCD.

As HFoVEMP can localize the affected side, we analyzed the results from both ears and related them to the surgical CT findings as shown in Figure 4. We have not found any case of doubtful response at HFoVEMP and SCD.

When just considering the SCD− and SCD+ type of CT scan with respect to positive and negative + doubtful responses, there is a significant relation between HFoVEMP and CT scan detection (chi-square = 18.1, *p* = 0.0001). The specificity of the HFoVEMP to detect a patient with SCD is 0.75 and its sensitivity is 0.81; the positive predictive value is 0.68, and the negative predictive value is 0.86.

### 3.4. SVIN and HFoVEMP

Both tests’ results were positive in 16 patients, negative in 6 and different in 8 (chi-square = 3.1, *p* = 0.07).

**Figure 5:**
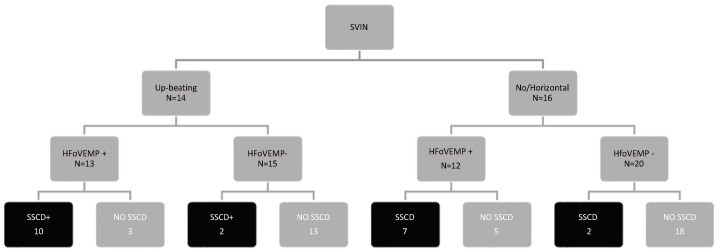
Combined results in SVIN and oVEMP and CT scan findings.

In Figure 5, we present the combined results by patient and ear. It is clear that up-beat SVIN will point to a SCD mainly when HFoVEMP is present, and when this is negative there is a high probability that it is not SCD. When SVIN is negative or is horizontal, HFoVEMP will be of help when it is negative; however, when there is an evoked response to 4 kHz, the probability of SCD is similar to a normal finding.

## 4. Discussion

The SCD diagnostic criteria [5] have been established in an attempt to homogenize the diagnosis of a disorder that, in many cases, may not be clear. This is not only because there are anatomical dehiscences that do not cause any symptoms, but also because there may be a radiological overestimation of SCD [2].

Apart from the characteristic symptoms of a third mobile window (bone conduction hyperacusis, sound-induced vertigo and/or oscillopsia, pressure-induced vertigo or pulsatile tinnitus) and the clinical findings in the form of nystagmus induced by sound or by pressure changes in the middle ear, some tests can help in the diagnosis of SCD, such as the pure tone audiometry (low-frequency negative bone conduction thresholds) or enhanced VEMP responses (low cervical VEMP thresholds or high ocular VEMP amplitudes). We have shown here that CT findings can be extremely heterogeneous between both sides when clinical suspicion is raised; we found it to be more frequent that one side is normal, and in the other there is clear dehiscent SSC, or it is near-dehiscent. To add complexity to this finding is the type of SVIN elicited that more frequently has a predominant vertical direction and is less often horizontal. This has limited further analysis of results, and a broader study is ongoing to detect the true specificity and sensitivity of SVIN.

Both oVEMP and cVEMP have been included in the diagnostic criteria for SCD, and it is recognized that oVEMP “has also been found to be highly sensitive and specific for SCD”, getting sensitivity and specificity greater than 90% from SCD [18,19,20].

It should be considered that unilateral SCD patients show greater sensitivity to high-frequency stimulation. In this sense, high frequency oVEMP has demonstrated its utility in the diagnosis of SCD [21]. This is because fluid displacement is increased in SCD when high vibration frequencies (500 Hz and higher) are applied. This fluid displacement is large enough to deflect the cilia [4,22,23].

The general principle of SVIN, in which the observed nystagmus has the same direction for 100 Hz vibration when stimulating both mastoids, beating away from the affected ear and with a horizontal and torsional component, is not applicable for SCD patients. A reverse SVIN beating towards the SCD ear is explained by the “imbalance principle” [23] because of the higher average neural activity in the vestibular nuclei on the affected side.

When unilateral SCD patients are studied, SVIN mainly shows torsional and horizontal components, and the vertical component (if present) is up beating, due to the stimulation of the superior canal [14].

In our work, we try to combine the results of two tests that have proven to be highly effective in detecting SCD. In the case of SVIN, its utility combined with other vestibular tests in the detection of vestibular entities has been demonstrated [13].

SVIN detection of SCD in our group of patients is 66% when SVIN has been described to be observed in between 82 and 100% of SCD patients [7,24]. It should be noted that we have not distinguished uni- or bilateral SCD in the global consideration for positive or negative SVIN. In total, 5/30 of our patients were considered as bilateral SCD. SVIN sensitivity in bilateral SCD is lower than in unilateral SCD because the SVIN acts as an indicator of vestibular asymmetry being better in these cases than the use of HFoVEMP that studies each side independently [14].

The presence of a positive SVIN, with an evident up-beating vertical component, and a positive HFoVEMP is highly suggestive of SCD. The SVIN vertical component is more often up-beating than down-beating [7].

On the other hand, when both tests are negative, or the HFoVEMP is negative and the SVIN shows a preferably horizontal component, the possibilities of finding SCD are limited. 

When HFoVEMP is positive but SVIN is negative or shows a horizontal component, it is not possible to conclude the presence of SCD. HFoVEMP testing is highly specific for the detection of SCD, and when the results are added to other tests, its sensitivity is improved [25,26].

The first mention of VIN in SCD was provided by Kramer PD and Zee D (1998) in an oral presentation of the XX at the Barany Society (Wurzburg 11–12 September 1998), and the first recording of VIN in a uSCD with a 2D device was published in 2005 [27]. Since then, the steps for improving the knowledge about the clinical implication of SVIN has been constant. The SVIN in SCD has been one of them.

## 5. Conclusions

Based on the results obtained, we conclude that SVIN, and in particular up-beating, is highly frequent in patients with SCD. However, it is not useful in itself to differentiate clear SCD from other less defined findings such as near-dehiscence.

When the results obtained in the SVIN are added to those of HFoVEMP, it not only improves the possibilities of detecting SCD, but also the affected side can be better located. HFoVEMP is a reliable tool for SCD detection, and it is possible to determine pure SCD from near-dehiscence findings in CT scans.

## Figures and Tables

**Table 1:** CT findings in the patients by ear. YES indicates superior semicircular canal dehiscence, and NEAR D, a near-dehiscence.

			Left Ear		Total
		NO	NEAR D	YES	
	NO	-	4	2	6
Right Ear	NEAR D	9	1	-	10
	YES	7	2	5	14
Total		16	7	7	30

**Table 2:** Nystagmus and parameters of nystagmus according to findings in temporal bone CT scan. N: number of patients; SPV: slow phase velocity of nystagmus; NO: normal temporal bone; NearSSCD: near-dehiscent superior semicircular canal; SSCD: dehiscent superior semicircular canal.

	Horizontal SVIN		Vertical SVIN	
Findings in TB	N	SPV (Mean ± SD)	N	SPV
**NO&NearSSCD**	1/13	2.9°s^−1^	5/13	1.7 ± 0.3
**NO&SSCD**	4/9	4.1 ± 2.3	4/9	1.9 ± 0.7
**SSCD&SSCD**	1/5	4.5	4/5	2.3 ± 0.8
**SSCD&NearSSCD**	0/2		0/2	
**NearSSCD&NearSSCD**	0/1		0/1	

## Data Availability

Not applicable.
